# Herbal remedy knowledge acquisition and transmission among the Yucatec Maya in Tabi, Mexico: a cross-sectional study

**DOI:** 10.1186/s13002-015-0022-6

**Published:** 2015-04-30

**Authors:** Allison L Hopkins, John Richard Stepp, Christopher McCarty, Judith S Gordon

**Affiliations:** Department of Family and Community Medicine, University of Arizona, Tucson, AZ USA; Department of Anthropology, University of Florida, Gainesville, FL USA; Department of Anthropology and Bureau of Economic and Business Research, University of Florida, Gainesville, FL USA

**Keywords:** Knowledge acquisition and transmission, Ethnobotany, Medicinal plants, Yucatan

## Abstract

**Background:**

Ethnobotanical knowledge continues to be important for treating illness in many rural communities, despite access to health care clinics and pharmaceuticals. However, access to health care clinics and other modern services can have an impact on the distribution of medical ethnobotanical knowledge. Many factors have been shown to be associated with distributions in this type of knowledge. The goal of the sub-analyses reported in this paper was to better understand the relationship between herbal remedy knowledge, and two such factors, age and social network position, among the Yucatec Maya in Tabi, Yucatan.

**Methods:**

The sample consisted of 116 Yucatec Maya adults. Cultural consensus analysis was used to measure variation in herbal remedy knowledge using competence scores, which is a measure of participant agreement within a domain. Social network analysis was used to measure individual position within a network using in-degree scores, based on the number of people who asked an individual about herbal remedies. Surveys were used to capture relevant personal attributes, including age.

**Results:**

Analysis revealed a significant positive correlation between age and the herbal medicine competence score for individuals 45 and under, and no relationship for individuals over 45. There was an insignificant relationship between in-degree and competence scores for individuals 50 and under and a significant positive correlation for those over 50.

**Conclusions:**

There are two possible mechanisms that could account for the differences between cohorts: 1) knowledge accumulation over time; and/or 2) the stunting of knowledge acquisition through delayed acquisition, competing treatment options, and changes in values. Primary ethnographic evidence suggests that both mechanisms may be at play in Tabi. Future studies using longitudinal or cross-site comparisons are necessary to determine the whether and how the second mechanism is influencing the different cohorts.

## Resumen en Español

**Antecedentes:**

El conocimiento de la etnobotánica continua siendo importante para el tratamiento de enfermedades en muchas comunidades rurales del mundo, a pesar de que en muchos casos exista acceso a centros de cuidados clínicos y farmacias. Sin embargo, este acceso a los centros de salud y otros servicios modernos, puede tener un impacto sobre la distribución del conocimiento de la etnobotánica médica. Muchos factores han demostrado estar asociados con distribuciones en este tipo de conocimiento. El reto de los sub-análisis reportado en este documento fue entender mejor la relación entre el conocimiento de remedio herbal y dos de estos factores, la edad, y la posición dentro de la red social, entre el Maya yucateco de Tabi, Yucatán.

**Métodos:**

La muestra consistió de 116 adultos maya yucatecos. Se utilizó un análisis de consenso cultural para medir la variación en el conocimiento de remedios herbales utilizando marcadores de aptitud, a través de una medición de concordancia entre participantes sobre un dominio. También se utilizó un análisis de redes sociales para medir la posición del individuo dentro de la red usando marcadores de grado, a través del número de personas que preguntaron a un solo individuo acerca de su conocimiento de remedios herbales. Por otro parte se utilizarán cuestionarios para obtener atributos personales relevantes, incluyendo la edad.

**Resultados:**

Los análisis revelaron una correlación positiva significante entre la edad y los marcadores de aptitud de conocimiento en medicina herbal para individuos de 45 años y menores, y sin ninguna relación para individuos mayores a 45 años. Se encontró una relación insignificante entre marcadores de grado y aptitud para individuos de 50 años y menores, y una correlación positiva significante para aquellos individuos mayores a 50 años.

**Conclusiones:**

Existen dos mecanismos posibles que podrían explicar las diferencias entre cohortes: 1) la acumulación de conocimiento en el tiempo; y/o 2) el retraso en el crecimiento de la adquisición del conocimiento a través de la adquisición tardía, compiten las opciones de tratamiento y los cambios en los valores. La principal evidencia etnográfica sugiere que ambos mecanismos podrían estar en juego en Tabi. Futuros estudios utilizando comparaciones longitudinales o entre sitios se consideran necesarios para determinar si se está influyendo sobre las diferentes cohortes, y como el segundo mecanismo lo está haciendo.

## Background

Ethnobotanical knowledge, including the knowledge of how to grow and process plants for housing, clothing, food, medicine, storage, and fuel, is important for survival and well-being in most subsistence farming communities [1-3]. For example, medical ethnobotanical knowledge can be used by community members to heal common illnesses when access to medical doctors or pharmaceuticals is limited or the side effects produced by medications are undesirable. Many rural communities in developing countries are increasingly obtaining access to modern services, including public schools and health care facilities, and experiencing greater integration into the market economy. These changes have the potential to impact the distribution of medical ethnobotanical knowledge among members of these communities.

Studies of medical ethnobotanical knowledge distribution have generally focused on determining the personal attributes associated with variation in knowledge. The attributes most commonly identified are age [4-8], gender [7,9-15], livelihood strategies [7,12,16,17], formal education [8,18-20], range and migration [8,21], religion [8,9], relative economic prosperity [8,12,17,22,23], lifestyle [8,9,24-27], and individual talents and motivation [7,24,28,29]. The explanations for why these personal attributes are associated with variation in ethnobotanical knowledge are often related to aspects of modernization. For example, several studies have found that adults who went to school for a greater numbers of years as children tend to have less ethnobotanical knowledge than those who went to school for less time [8,18-20]. Explanations for this difference include: adults with more schooling spent less time interacting directly with the natural environment during their childhood; and they may have less direct dependency on the environment as adults through increased non-farming job opportunities and shifting values than those who went to school for less time. This pattern may not always be the case, as it is also possible that schooling could increase ethnobotanical knowledge by creating the potential for people to utilize written sources of information [30].

Social networks are the structure through which ethnobotanical and other types of cultural knowledge is transmitted. In the past decade, researchers have started to use network concepts to understand patterns in natural resource use, knowledge, and management [31-36]. Nevertheless, only a few studies have mentioned the potential influence of relational variables on herbal knowledge transmission and distribution [21,37], and only one has linked relational variables to individual medicinal plant knowledge variation [38].

We carried out a study in Tabi, Yucatan, Mexico, a Yucatec Maya community where people have access to, and utilize to varying degrees, the public schools, modern health care facilities, and the market economy. The study was designed to respond to the question, “to what extent does individual network position explain variation in herbal knowledge across households in Tabi, independent of attribute characteristics of the individual?” Our approach was unique, not only because we focused on relational variables, but also because we used a more comprehensive approach than most researchers in this area by controlling for the influence of attribute variables that are known to be associated with herbal remedy knowledge. The main outcome of this study was that the individual’s position within the herbal knowledge exchange network was positively associated with herbal remedy knowledge when analyzed individually, but when the attribute variables were added, age masked all other associations, including network position [39,40].

Following the example of researchers working in other domains of ethnobotanical knowledge, we carried out an age cohort sub-analysis to further understand the relationship between age and herbal remedy knowledge [25,41]. We also carried out an age cohort sub-analysis with individual position within the herbal remedy knowledge exchange network. In this paper, we report on the results of these two sub-analyses.

## Methods

### The setting

The study took place from April 2007-April 2008 in Tabi, a small Yucatec Maya rural village located in the central part of the state of Yucatan, Mexico. The economy of the community is mostly based on subsistence agriculture, although increasingly the youth are migrating to the regional cities to perform low-skilled jobs. The communally held land (known as *ejido*) surrounding the village is characterized by low deciduous forest [42], which is utilized in the process of swidden agriculture practiced by most farmers. There is a public kindergarten, elementary school (grades 1–6), and middle school (grades 7–9) located in Tabi. The nearest high school (grades 10–12), which is also state-run, is located 11 kilometers from Tabi in Sotuta, the municipal center. Bilingualism is common in the community (89%) with only 9.5% the population monolingual in Yucatec Maya, and 1.5% in Spanish [43]. The massive Catholic Church that dominates the main plaza dates back to the 1700s. Smaller Protestant churches have been built in Tabi over the last several decades as an increasing number of people are moving away from the Catholic Church. Access to modern medicine has also increased during the same time period. In the late 1980s, a small, state-run health care clinic with a visiting physician was established in the community, and then in 2002, a yearly rotating medical resident from one of the medical schools was permanently installed in the clinic. Prescription pharmaceuticals are available at the state-run clinic, and over-the-counter pharmaceuticals at one of the six privately-owned corner stores in the community.

### Type of participants involved

The village is comprised of 698 people living in 122 households. The study design was based on households, and one adult from almost every household in Tabi participated in the study (n = 116). The adult selected was the most knowledgeable about medicinal plants in the household as determined by members of the household. In households where no one was considered knowledgeable, the participant was randomly chosen. Slightly more than half of the participants were female (54.3%), and ranged in age from 16 to 87, with a mean age of 46.4 (SD = 17.2) (Table 1). Although few participants received no formal education (15.5%), only 6.8% went on to middle school, and 30.2% were illiterate.

**Table 1:** **Participant demographics (n = 116)**

**Variable [Unit]**	**Value**
**Age** in years [Mean (SD)]	46.4 (17.2)
**Gender** [Percent]	
Male	45.7
Female	54.3
**Literate in Spanish** [Percent]	
Yes	69.8
No	30.2
**Education** [Percent]	
No formal schooling	15.5
Completed grades 1-3	46.5
Completed grades 4-6	31.1
Completed grades 7-9	6.8
**Religion** [Percent]	
Agnostic/Atheist	20.7
Catholic	45.7
Protestant	33.6
**Livelihood** [Percent]	
Homemaker	54.3
Full-Time Subsistence Farmers	31.9
Subsistence Farmers and Temporary Wage Laborers	12.1
Full-Time Wage laborers	1.7
**Places of residence** [Percent]	
Only Tabi	81
Other regional small towns	6
Regional cities (Merida or Cancun)	13
**Farthest Distance Travelled** in Kilometers as the Crow Flies [Mean (SD)]	254.5 (570.9)
**Relative lifestyle scale** [Percent]	
0 (Very traditional lifestyle)	1.7
1	3.4
2	26.7
3	22.4
4	24.1
5	19.8
6 (Very modern lifestyle)	1.7
**Relative economic prosperity scale** [Percent]	
1 (Very low relative economic prosperity)	8.6
2	31.9
3	34.5
4	20.7
5 (Very high relative economic prosperity)	4.3
**Interest in medicinal plants** [Percent]	
Very interested and interested	93.9
Neutral	0.9
Very uninterested and uninterested	5.2
**Used most to treat common illness** [Percent]	
Medicinal Plants	69
Pharmaceuticals	30.2
God’s Will	0.9
**General course of action for common illness** [Percent]	
Treat the illness at home	66.4
Visit a family member or acquaintance to treat the illness	3.4
Visit a traditional healer to treat the illness	0.9
Visit a conventional medical doctor to treat the illness	28.4
Have faith in God’s will	0.9

The most common religion in Tabi continues to be Catholicism (45.7%); however, many people had joined one of the Protestant churches in the community (33.6%) or had no religious affiliation (20.7%). All of the women were homemakers who also care for the small livestock raised and horticultural crops grown within the area surrounding their houses. The majority of the men (69.8%) dedicated all their work time to tending the agricultural fields and large livestock on *ejido* land away from the home. Some men (26.4%) primarily performed temporary wage labor, and tended their fields the rest of the time, and only a very few (3.8%) were employed as full-time wage laborers. Thus, most of the participants spent much of their day outside in nature.

The majority of participants (81%) had lived in Tabi their entire lives. Of the people who lived elsewhere, most lived in Merida or Cancun (13%), the two largest regional cities; the remaining few (6%) lived in other small towns on the Yucatan Peninsula. The average distance people from Tabi had travelled as the crow flies is 254.5 kilometers. The farthest the majority of people travelled were to Merida (40.5%) or Cancun (27.6%). Of those who had travelled farther, the most common destination was Mexico City (5%). Two participants travelled to the United States and Canada as guest farm workers.

Almost three-quarters of the participants practiced a mix of traditional and modern lifestyles as determined by a series of items assessed through participant observation, and identified by participants as key indicators of a more modern lifestyle, including: consumption of leavened bread; occasional or frequent purchasing of machine-made tortillas; preference for speaking Spanish with children; preference for speaking Spanish with other adults; preference for wearing Western clothes; and ownership of a bed (see [44]). Based on a series of economic indicators assessed through participant observation, and identified by participants (including ownership of a hammock, television, stereo, refrigerator, and/or stove), the majority of participants (87.1%) had moderate economic prosperity. Almost all participants reported an interest in medicinal plants (94%). Over three-quarters of participants (88.8%) had used medicinal plants to treat their children, and over two-thirds of participants (69%) had used medicinal plants more often than pharmaceuticals (30.2%) to treat common illnesses. Additionally, most respondents (66.4%) reported treating common illnesses at home.

### Data collection and analysis

This was a cross-sectional study, meaning the variables were measured at one point in time among one group of people [45]. This research project was approved by the University of Florida Institutional Review Board on April 3, 2007 (IRB protocol number 2007-U-0259), and verbal informed consent was received from all participants prior to participating in the study per IRB approval. Multiple data collection and analysis techniques were utilized in this study to address our five objectives, including cultural consensus analysis (CCA), social network analysis (SNA), and surveys and statistical analyses [39].

Our first objective was to assess variation in herbal remedy knowledge in Tabi. First, we administered an herbal remedy knowledge questionnaire, and then we ran CCA. Typically CCA is a two part process. The first is a free-listing task asking respondents in the cultural context to list items, in this case herbal remedies consisting of a plant and an illness the plant can help treat. We did this task with 40 respondents in Tabi. The second part of CCA is a systematics survey asking respondents about a subset of remedies listed by the first group. For our study the survey developed in the second step consisted of 43 questions in the format “Can *plant x* cure *illness y*?”. We created the questionnaire by substituting *plant x* and *illness y* in approximately half of the questions in plant and illness combinations that had been free listed by at least 2 out of 40 people from the first step. The other half of the questions were plant and illness combinations that were not known remedies in Tabi or in the Yucatec Maya ethnobotanical literature. These items were added because CCA is sensitive to greatly unequal proportions of yes and no responses [46]. For each question, the response choices were “yes”, “no”, or “uncertain”. In cases where the individual was uncertain, the response was imputed by a simulated coin toss since CCA operates under the assumption that respondents will guess when uncertain [46,47].

We analyzed the data by running a CCA in UCINET, which measured individual variation in agreement of botanical remedies among households within Tabi and determined if there was one common culture of herbal remedy knowledge in Tabi [47,48]. The data from Tabi did not violate any of the model’s assumptions, thus there is one common culture of herbal remedy knowledge, and the first factor values represent individual competence scores for that knowledge [39]. A more detailed description of this methodology and some of the results have been previously published [39,40]. In summary, the average competence scores was 0.64 (SD = 0.20) with a range from a low level of competence at 0.08 to a high level of competence at 0.95. To put this in perspective, a competence level of 1.0 would mean that a participant’s responses to herbal remedy survey matched the group consensus in responses. A score of zero means they never agreed with the group. The relatively large standard deviation and the wide range in competence scores indicate that across households in Tabi, there is a great deal of variation in agreement about botanical remedies.

Our second objective was to describe the herbal remedy knowledge network in Tabi, and determine participants’ positions within that network. First, we asked each participant: “Have you asked *person x* about herbal remedies?” substituting the names of each of the other 115 participants for *person x*. Then, we analyzed the relational data using UCINET and NetDraw [48]. UCINET produced several overall measures of network structure and individual position within the network (centrality measures), and NetDraw provided a visualization of the whole network. Pearson’s correlations were performed to assess the relationship between competence scores and relational measures. We have reported on this methodology and some of the results in a previous publication [39]. In this paper we present new data and a novel focus specifically on in-degree, the only centrality measure that was correlated with competence scores (*r* = 0.28, *p* < 0.01, *N* = 116). In-degree represents the number of individuals who asked a participant about medicinal plant remedies.

Our third objective was to measure individual attributes of the participants. Attributes of network members can be used to try to explain their network position. These attributes were collected by administering a survey. The questions in the survey were informed by previous research on intra-cultural variation in traditional ecological knowledge, and ethnographic information obtained during the first six months of fieldwork. The questions focused on age [4-8], gender [7,9-15], livelihood strategies [7,12,16,17], level of formal education [8,18-20], religious affiliations [8,9], economic prosperity [8,12,17,22,23], lifestyle [8,9,24-27], range and migration [8,21], treatment preferences and perceived interest in herbal medicine [7,24,28,29] since these are factors that have been associated with knowledge variation. Descriptive statistics were performed and the results are presented in Table 1. Pearson’s correlations and ANOVAs were performed to assess the relationship between competence scores, and continuous and categorical attribute variables, respectively. A general description of the results and a discussion of the inferential findings have been previously reported [44].

Our fourth objective was to test the hypothesis that greater individual herbal remedy knowledge is positively associated with the position of individuals within the botanical remedy knowledge network; that is, the more people report talking to someone about remedies the more that person tends to agree with the group. The alternative, a lack of association, would suggest that people report talking about remedies with those who are not experts. We did this test by running regressions using permutation tests with competence scores as the outcome variable, and individual centrality measures and the attribute variables as the explanatory variables [49]. Description of these analyses and results are previously published [39], but in summary the model predicted 26.1% of the variation in competence scores (*F* = 3.34, *p* = 0.04, *N* = 116) and age masked the association between competence scores and all other variables including in-degree (*B* = 0.01, *p* < .01, *N* = 116). We then ran a Pearson’s correlation between age and in-degree and determined that they are positively and strongly associated (*r* = 0.48, *p* < 0.01), suggesting that not only are older individuals more knowledgeable about herbal remedies, but they are also a source of information about herbal remedies for more people.

Our fifth objective, and the primary focus of this paper, was to determine if the relationship between age and knowledge identified in objective four was consistent across age groups. We found that overall competence increases with age, but this pattern may not be the same for all age cohorts. If the pattern differs by age group and is associated with an event or major change in the community, then it provides evidence for changes in acquisition patterns which may be resulting in knowledge loss [25,41]. We divided the participants into two different age cohorts, and ran Pearson’s correlations between competence scores, in-degree and age variables. The age cohorts were 16–45 and 46–87 for age and competence, and 16–50 and 51–87 for age and in-degree. We also conducted ethnographic interviews with a subsample of 20 participants related to the findings to foster interpretation. The interviews were focused on community history, and patterns in acquisition and transmission.

## Results and discussion

### Age and competence

**Figure 1:**
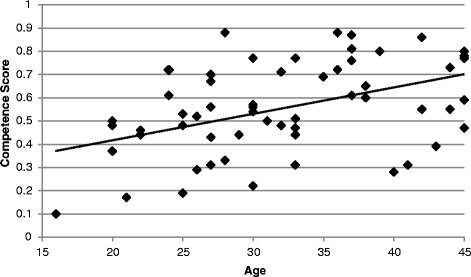
Relationship between competence scores and age. There is a positive association between competence score (agreement about medicinal plant remedies) and age (*r* = 0.46, *p* < 0.01, N = 59) for individuals from 16 through 45 years of age.

**Figure 2:**
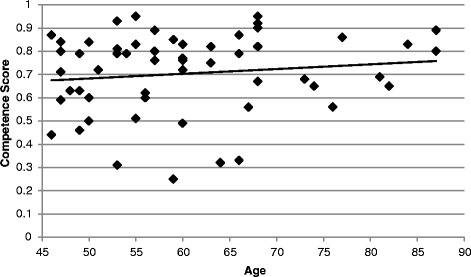
Relationship between competence score and age. There is no association between competence score (agreement about medicinal plant remedies) and age (*r* = 0.13, *p* = 0.32, N = 57) for individuals from 46 through 87 years of age.

The cohort analysis revealed that there was a positive correlation between age and competence scores for individuals 45 and younger (*r* = .46, *p* < 0.01, N = 59), but no relationship for individuals 46 and older (*r* = 0.13, *p* = 0.32, N = 57) (Figures 1 and 2). In ethnographic interviews, participants generally explained that they learned what they know about medicinal plants *after* they had children, with 40% reporting they started learning about herbal remedies shortly after they got married, 40% after they had their first child, and 10% once their children started getting sick. Only 10% reported that they learned about medicinal plants as children. Prince [50] also found increased motivation among the Luo in Kenya once they had children. These findings provide evidence that the relationship between age and competence scores in the younger cohort is at least partially explained by accumulation of knowledge.

Another factor that could be related to the difference between age cohorts is a decreased interest in learning about medicinal plants as a result of modernization and related lifestyle changes. Since the end of the Mexican Revolution (~1920), the Mexican government has been expanding basic services to rural communities. Around 50 years ago, these services finally reached Tabi. The first change was the establishment of the elementary school around 1955. During the 1960s, visiting doctors began to come to the village weekly. Many additional changes occurred in Tabi during Victor Cervera Pacheco’s governorship of the Yucatan (1984 to 1988), including installation of reliable electricity and potable water, paving of the road between Sotuta (the municipal capital) and Tabi, and establishment of the *Servicios de Salud de Yucatán* (SSY) health center. In 1994, the road to Yaxcaba (the municipal capital of the municipality just to the east of Tabi) was paved, and two years later, a twice daily bus route began transporting people to and from Merida. Internships by medical residents in the SSY health center became a requirement in 2002, and the interns began offering free daily medical services for community members insured under *Seguro Social* or *Seguro Popular*.

These gradual modernization efforts in Tabi led to an intensification of inter-cultural contact, economic integration, and westernization, and may have influenced the quantity and quality of information acquired by younger individuals regarding traditional medicine. If this is the case, these changes may be related to the variation we found between age cohorts in competence scores. Zent [25] found a similar pattern of knowledge among the Piaroa in Venezuela. He determined that the difference between the two cohorts corresponded to their relocation and ensuing lifestyle change. Voeks and Leony [8] saw a striking difference in knowledge levels between the 61–70 age group and 71–80 age group in Lençois, Brazil. Voeks and Leony [8] argue that the extreme difference in knowledge was a result in the shift from using traditional medicine as the only illness survival strategy to having multiple options for treated illnesses.

Changes in lifestyle may be affecting the timing of knowledge transmission as well. There is some evidence in populations where traditional peasant lifestyles are the norm that the positive correlation between age and medicinal plant knowledge is not as strong or nonexistent [26,51]. In the case of the Hoti of Venezuela, a group with relatively low levels of acculturation, medicinal plant knowledge acquisition starts in early childhood and increases until 18–28 years of age [52]. These studies provide evidence that in less-acculturated groups, the bulk of basic ethnobotanical knowledge is learned in childhood, and knowledge accumulation slows or stops in adulthood. Tzotzil Maya children living in a rural community know more plants earlier than their counterparts living in the town center [53]. In Tabi, almost all of the medicinal plant remedy knowledge is learned in adulthood, suggesting that lifestyle changes may be affecting transmission practices. There were only two individuals from the subsample who learned the bulk of what they know about medicinal plants as children: a 77-year-old woman and a 59-year-old man.

Changes in lifestyle may also be affecting the modes of cultural transmission. There is some evidence that when knowledge about medicinal plants is acquired during childhood the transmission mode is generally vertical (i.e., from parent to child) [50,51,54] and/or concerted (i.e., from older members of the social group to younger members of the group) [55]. In Tabi, most participants (66%) were asked by relatives for information about medicinal plants. However, more than a fifth of the people (21%) asked about medicinal plants were identified as healers or particularly knowledgeable about medicinal plants. A little over one-tenth (13%) of the people asked for information were neighbors, friends, and acquaintances. Of those who were asked about medicinal plants, 58% were from an older generation than the person asking, 29% from the same generation, and, surprisingly, 11% from a younger generation. Thus, a change in timing of acquisition may lead to a shift from primarily vertical and/or concerted transmission to multiple modes of transmission including vertical, horizontal (i.e., between individuals of the same generation), one-to-many (i.e., healer to others), and concerted [56]. This shift in prevalence of different modes of cultural transmission may also help explain the relationship between age and competence in that each mode varies in the degree with which knowledge is shared between individuals in a group [54].

### Age and position in network

**Figure 3:**
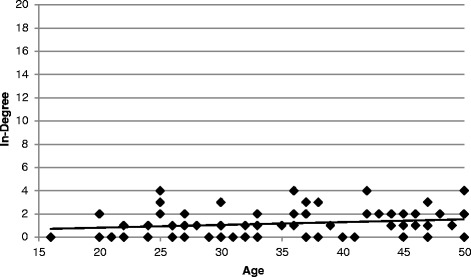
Relationship between in-degree and age. There is no association between in-degree and age for individuals from 16 through 50 years of age (*r* = 0.19, *p* = 0.11, N = 72).

**Figure 4:**
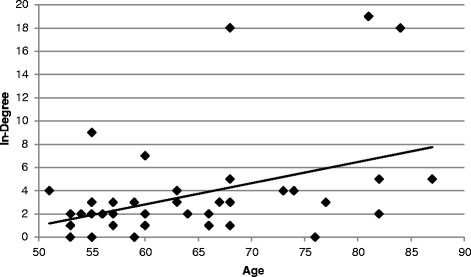
Relationship between in-degree and age. There is a positive association between in-degree and age for individuals from 51 through 87 years of age (*r* = 0.42, *p* < 0.01, N = 44).

A cohort analysis between age and in-degree showed no relationship between the two variables in the participants ages 16–50 (*r* = 0.19, *p* = 0.11, N = 72), and a positive relationship from 51 to 87 years of age (*r* = 0.42, *p* < 0.01, N = 44) (Figures 3 and 4). This pattern corresponds with ethnographic findings that there is a general respect for Tabi elders and the herbal remedy knowledge they possess within the community. Older individuals have gained valuable experience using medicinal plants while raising their children. That knowledge backed by experience is sought out by individuals with young children who have limited experience with herbal treatments. Additionally, it fits with a widespread belief that younger people know little about medicinal plants. After finishing an interview with a participant, they would frequently ask us who we were going to visit next. If we were speaking with an older individual and we were going to visit a younger member of the community, they would often say something regarding the lack of knowledge of the person we intended to interview. Then they would suggest two or three older individuals that we should interview. Also, on several occasions younger individuals suggested we interview older community members instead of themselves, citing their lack of knowledge compared to the older individuals. Lastly, older individuals have more spare time to help treat illnesses when they occur. One mother explained to us that although she is very interested in medicinal plants, she found it difficult to learn about them with all the demands of raising several young children. Instead she chose to take her sick children to her mother-in-law for treatment. However, she recognized the need for her to learn the remedies so that she can use them and pass them on to her children after her mother-in-law dies. This example highlights the challenges, and potential risks, of learning about herbal remedies as an adult.

## Conclusions

The first age cohort and knowledge sub-analysis revealed a positive correlation between age and herbal remedy knowledge among the younger cohort, and no association among the older cohort. There are two possible mechanisms that could account for the differences between cohorts. The first is that younger individuals are accumulating the knowledge they need for their basic herbal remedy toolkit, whereas older individuals have already acquired this knowledge. The second is that individuals in the younger cohort are learning less medical ethnobotanical knowledge because of: increased access to modern services (e.g. schools and health care clinics); reduced traditional learning opportunities; changed values; and greater treatment options. Ethnographic evidence suggests that both mechanisms may be at play in Tabi. The opposite pattern was observed with age cohort and network position sub-analysis with no association between age and in-degree among the younger cohort, and a positive correlation among the older cohort. This pattern was also supported by the ethnographic evidence.

The modernization processes affecting knowledge acquisition and transmission are not unique to Tabi, and communities like Tabi are rapidly becoming the norm. This suggests that the findings in this study may be representative of other communities where people are predominantly subsistence farmers, bilingual in their native and national languages, have access to modern medicine, and have increasing contact with the national culture. Comparisons between already existing studies suggest that ethnobotanical knowledge is acquired earlier in life and there is less variation between individuals in communities with less disruption of daily life by external processes than in communities with more changes [19,25,51,52,57,58].

Changes in ethnobotanical knowledge can be more fully understood by designing and carrying out studies that use systematic approaches to data collection and analysis. There are several approaches that are particularly well suited to facilitate our understanding of cultural knowledge change, including: 1) comparing knowledge variation in communities with varying degrees of modernization or participation in market economy but have otherwise similar characteristics [25,52,53], 2) comparing knowledge between migrants and those continuing to live in their home country [59] and 3) by carrying out longitudinal studies to assess changes in knowledge over time in the same location u. In cases where these types of studies are not feasible, an alternative strategy is to standardize a methodology, such as the one used in this study, which researchers can use to systematically measure knowledge variation across studies and sites. More research using these approaches is needed to develop and test theories regarding ethnobotanical knowledge acquisition, transmission, variation, dynamism, and loss.

## Abbreviations

IRB: Institutional Review Board
CCA: Cultural consensus analysis
SNA: Social network analysis
SSY: Servicios de Salud de Yucatan (Health Services of Yucatan)
